# Electrocardiogram-Gated 320-Slice Multidetector Computed Tomography for the Measurement of Pulmonary Arterial Distensibility in Chronic Thromboembolic Pulmonary Hypertension

**DOI:** 10.1371/journal.pone.0111563

**Published:** 2014-11-03

**Authors:** Hajime Kasai, Toshihiko Sugiura, Nobuhiro Tanabe, Yoriko Sakurai, Misuzu Yahaba, Yukiko Matsuura, Ayako Shigeta, Naoko Kawata, Seiichiro Sakao, Yasunori Kasahara, Koichiro Tatsumi

**Affiliations:** 1 Department of Respirology, Graduate School of Medicine, Chiba University, Chiba, Japan; 2 Department of Advanced Medicine in Pulmonary Hypertension, Graduate School of Medicine, Chiba University, Chiba, Japan; Pulmonary Medicine, China

## Abstract

**Background:**

We aimed to study whether pulmonary arterial distensibility (PAD) correlates with hemodynamic parameters in chronic thromboembolic pulmonary hypertension (CTEPH) using electrocardiogram (ECG)-gated 320-slice multidetector computed tomography (MDCT).

**Methods and Findings:**

ECG-gated 320-slice MDCT and right heart catheterization (RHC) was performed in 53 subjects (60.6±11.4 years old; 37 females) with CTEPH. We retrospectively measured the minimum and maximum values of the cross sectional area (CSA) of the main pulmonary artery (mainPA), right pulmonary artery (rtPA), and left pulmonary artery (ltPA) during one heartbeat. PAD was calculated using the following formula: PAD = [(CSAmaximum−CSAminimum)/CSAmaximum]×100(%). The correlation between hemodynamic parameters and PAD was assessed. Mean pulmonary arterial pressure (mPAP) and pulmonary vascular resistance (PVR) were 40.8±8.7 mmHg and 8.3±3.0 wood units, respectively. PAD values were as follows: mainPA (14.0±5.0%), rtPA (12.8±5.6%), and ltPA (9.7±4.6%). Good correlations existed between mainPAD, with mPAP (r = −0.594, p<0.001) and PVR (r = −0.659, p<0.001). The correlation coefficients between rtPAD and ltPAD with pulmonary hemodynamics were all lower or equal than for mainPAD.

**Conclusions:**

PAD measured using ECG-gated 320-slice MDCT correlates with pulmonary hemodynamics in subjects with CTEPH. The mainPA is suitable for PAD measurement.

## Introduction

Chronic thromboembolic pulmonary hypertension (CTEPH) can result from either single or recurrent pulmonary embolism arising from deep vein thrombosis [1]. When resistance increases in the pulmonary circulation, the pulmonary artery becomes more distended and less distensible [2]. A study of pulmonary arterial compliance (Cp) suggested that pulmonary arterial distensibility (PAD) decreases with rising pulmonary arterial pressure [3]. PAD is customarily measured using magnetic resonance imaging (MRI) or transthoracic echocardiography, and more recently, computed tomography (CT) [2], [4]–[6]. In addition, although PAD has been correlated with prognosis, exercise tolerance, and pulmonary hemodynamic data among patients with pulmonary hypertension (PH) [6]–[10], the etiology of PH varies, and PAD varies between the main pulmonary artery (mainPA) and the right pulmonary artery (rtPA).

State-of-the-art multidetector CT (MDCT) scanners are now widely available and permit rapid acquisition of a thin-slice dataset, even in breathless patients [11]. A 320-slice MDCT affords 16 cm craniocaudal coverage and volumetric imaging of the entire heart with only a single gantry rotation [12]. In addition, a series of two gantry rotations (double volume scan) in combination with an electrocardiogram (ECG), i.e. ECG-gated 320-slice MDCT, can acquire simultaneous images of the pulmonary arteries and entire heart. Furthermore, because of advances in CT technology, ECG-gated MDCT has been used to image the heart with high spatial and temporal resolution [13].

We hypothesized that ECG-gated 320-slice MDCT could be used for less invasive and simultaneous evaluation of the pulmonary artery and hemodynamic data in the diagnostic assessment of patients with CTEPH. This study therefore aimed to evaluate the relationship between PAD of mainPA, rtPA, and the left pulmonary artery (ltPA) on ECG-gated 320-slice MDCT and hemodynamic data measured by right heart catheterization (RHC).

## Materials and Methods

### Study population

The study group consisted of consecutive patients with a high clinical suspicion of CTEPH who underwent ECG-gated 320-slice MDCT and RHC from September 2009 to December 2013. The study was approved by the ethics committee of Chiba University (approval number 826). Written informed consent to use their examination results in the future study was obtained from each patient before CT and RHC. Patients with complications including left heart disease, connective tissue disease, severe chronic obstructive pulmonary disease (GOLD Stage III–IV), and severe interstitial pneumonia were excluded.

### Enhanced ECG-gated 320-slice CT

All CT scan results were obtained retrospectively from enhanced ECG-gated volume scanning using 320-slice MDCT (Aquilion One, Toshiba Medical, Tochigi, Japan) with a slice thickness of 0.5 mm and 0.35 sec/rotation. To acquire simultaneous images of the pulmonary arteries and the entire heart, an axial series of two gantry rotations in a craniocaudal direction was performed (double volume scan). The resulting dual volume data sets were automatically stitched. Because the most cranial and caudal parts of each volume data set (both 1.6 cm) were not used to create images, the effective scan length was 25.6 cm. The tube voltage and current were set at 120 kV and 580 mA, respectively, with tube current dose modulation. Using a mechanical injector (Dual Shot, Nemoto Tokyo, Japan), 100 mL of contrast media (Iomeron 350 mg/ml, Eisai, Tokyo, Japan) was injected at 3.5 mL/s. Then a saline–contrast mixture was injected: 40 mL of contrast media at 2.0 mL/s, and 30 mL saline at 1.5 mL/s. Time-resolved (per second) single-section CT scans were acquired at the level of the bifurcation of the pulmonary artery without a breath hold. Ascending aortic time-resolved attenuation was then measured using the time-attenuation evaluation program accessible on the scanner. When the CT values in the ascending aorta had increased to 200 Hounsfield units (HU), we began the scan, and the subject was asked to hold the breath.

### PAD on ECG-gated 320-slice CT

**Figure 1:**
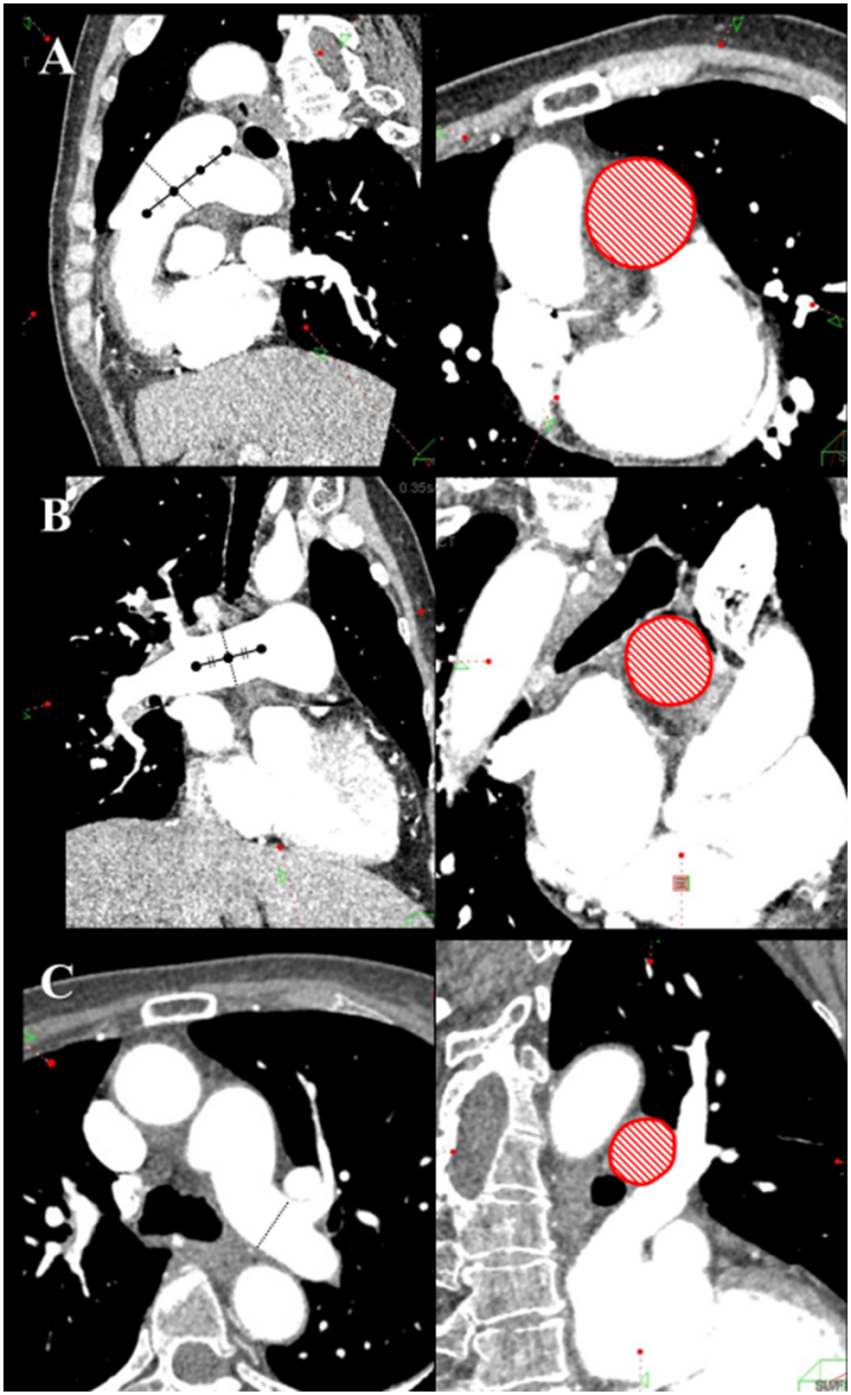
Measurement position of the cross-sectional area (CSA) of the pulmonary artery. (A) CSA of the main pulmonary artery was measured at the one-third position on the pulmonary valve side between the pulmonary valve and the bifurcation of the pulmonary artery. (B) CSA of the right pulmonary artery was measured at the central position between the bifurcation of the pulmonary artery and the first branch. (C) CSA of the left pulmonary artery was measured just before the left A3 branch.

The CT images were reconstructed at 5% intervals from 0% to 95% of the R–R interval. They were sent to a workstation (Ziostation2; Ziosoft Incorporated) that created cine images of the pulmonary arteries. The cross-sectional area (CSA) of the mainPA (mainPA-CSA) was measured at the one-third position of the pulmonary valve side between the pulmonary valve and the bifurcation of pulmonary artery (Fig. 1A). The CSA of the rtPA (rtPA-CSA) was measured at the central position between the bifurcation of the pulmonary artery and the first branch (Fig. 1B). The CSA of the ltPA (ltPA-CSA) was measured just before the left A3 branch (Fig. 1C). Each CSA position was determined after 30% of the cardiac cycle and adjusted to be perpendicular to the long axis on both axial and coronal views. The minimum and maximum values of each CSA during the cardiac cycle were measured at mediastinal window settings (level, 0 HU; width, 300 HU). When the vessel contained a mural thrombus, we measured the CSA of the blood vessel lumen by using the inner side of the vessel wall as the boundary, ignoring the thrombus. All CSA measurements were performed by two independent readers blinded to the patient’s identity.

**Figure 2:**
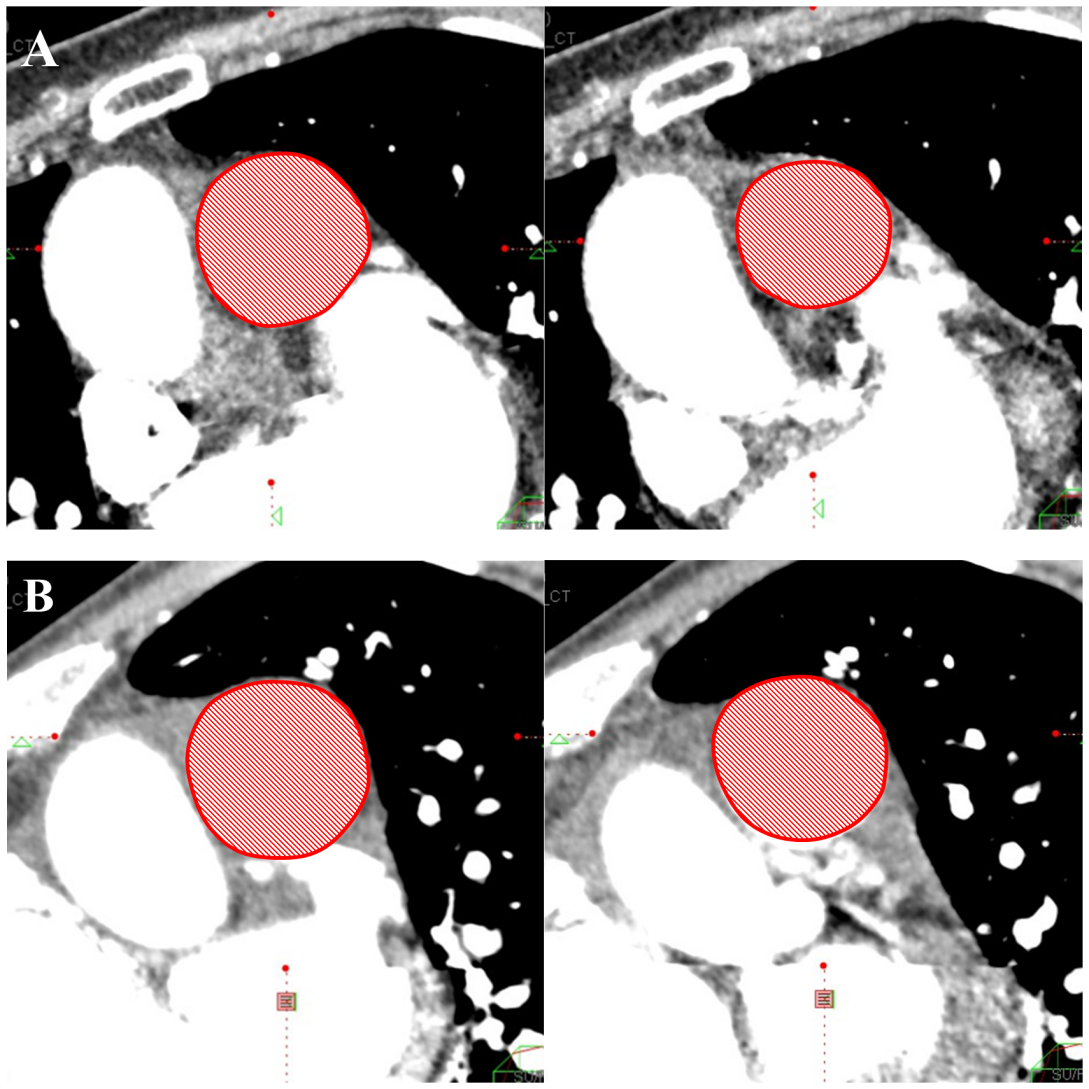
Assessment of main pulmonary arterial distensibility (mainPAD). (A) 78-year-old woman (mean pulmonary arterial pressure [mPAP], 34 mmHg). The maximum main pulmonary artery (mainPA)-cross sectional area (CSA) was 10.1 cm^2^, and the minimum was 8.2 cm^2^. The mainPAD was 18.7%. (B) 61-year-old man (mPAP, 44 mmHg). The maximum mainPA-CSA was 10.6 cm^2^, and the minimum was 9.6 cm^2^. The mainPAD was 8.7%.

PAD of the mainPA, rtPA, and ltPA (mainPAD, rtPAD, and ltPAD, respectively) were calculated using the following equation, and expressed as percentage variation: PAD = [(CSAmaximum−CSAminimum)/CSAmaximum]×100 (Fig. 2) [14].

### RHC

A 7.5-French gauge Swan–Ganz thermodilution catheter (Edwards Lifescience, USA) was used via a jugular approach. Pressure measurements were taken from the superior vena cava, right atrium, right ventricle, and mainPA at end-expiration. The zero point was defined as mid-thoracic. Cardiac output (CO) was determined using the thermodilution method by averaging a minimum of three measurements. Left-to-right shunting was ruled out by oximetry. Pulmonary vascular resistance (PVR) in Wood units (WU) was calculated using the equation: PVR = [mean pulmonary artery pressure (mPAP)−pulmonary artery wedge pressure (PAWP)]/CO. Cp was calculated by the ratio between stroke volume (SV) and pulmonary arterial pulse pressure (PAPP).

The PAD measured by enhanced ECG-gated 320-slice MDCT were assessed against the following values obtained by RHC: mPAP, systolic pulmonary arterial pressure (sPAP), diastolic pulmonary arterial pressure (dPAP), PAPP, PVR, CO, cardiac output index (CI), SV, and Cp.

### Clinical worsening analysis after sildenafil

The 13 patients who were administered sildenafil were followed for a mean of 24.8±11.1 months (range, 5–39 months). Time to clinical worsening, indicated by death or hospitalization due to right heart failure, between two groups stratified by median value of main PAD (14%) was compared.

### Statistical analysis

The Bland–Altman analysis was used to determine the inter- and intra-observer variation in PAD measurements. Pearson’s correlation was used to assess the correlation between hemodynamic data from RHC with PAD obtained using ECG-gated 320-slice MDCT. Correlation analysis of parameters showing a non-normal distribution was carried out using the Kendall tau rank correlation. All results are expressed as the mean ± standard deviation, unless otherwise indicated. A P value<0.05 was considered statistically significant. All statistical analyses were performed using JMP 9.0 software (Cary, North Carolina, USA).

Survival curves were estimated using the Kaplan-Meier method, and the log-rank test was used to compare survival curves according to clinical characteristics.

## Results

The study group comprised 53 consecutive patients (age, 60.6±11.4 [SD] years; females, 37) with CTEPH diagnosed by RHC and CT and/or pulmonary angiography. Among them, 24 patients underwent these examinations at the time of the diagnosis, and 29 patients underwent the examinations during follow-up after the diagnosis. Tables 1 and 2 show the baseline characteristics of the patients. The interval range between CT and RHC was two to sixteen days. All patients had perfusion defects (all were larger than segmental), with normal ventilation scans. CTEPH was confirmed by pulmonary angiography in all cases.

**Table 1:** Baseline clinical information of patients with CTEPH (n = 53).

Baseline parameters	
Age (years)	60.6±11.4
Gender (female/male)	37/16
Body surface area (m^2^)	1.60±0.21
CT-RHC Interval (day, min-max)	2–16
Oxygen therapy	40 (1–6 L/min)
Months from onset of symptoms to CT, median (range)	15 (4–306)
Vasodilators	
Oral prostaglandin I2	12
PhosphodiesteraseV inhibitor	15
Endothelin antagonist	8
Monotherapy	14
Double combination therapy	11
Triple combination therapy	0

Data are presented as mean±SD.

**Table 2:** Hemodynamic data of patients with CTEPH (n = 53).

Hemodynamic data	Mean ± SD	Median (min–max)
mPAP (mmHg)	40.8±8.7	41 (25–68)
sPAP (mmHg)	72.4±17.1	70 (39–126)
dPAP (mmHg)	21.3±6.2	22 (7–35)
PAPP (mmHg)	51.1±14.5	50 (27–102)
PVR (wood units)	8.3±3.0	8.4 (2.3–14.9)
CVP (mmHg)	6.1±3.3	5 (0–18)
PAWP (mmHg)	8.2±3.2	8 (4–15)
CO (L min^−1^)	4.1±0.8	4.0 (2.7–6.2)
CI (L min^−1^ m^−2^)	2.6±0.4	2.5 (1.6–3.8)
SV (ml)	59.8±17.1	54.0 (35.5–102.5)
Cp (ml/mmHg)	1.3±0.5	1.2 (0.6–3.1)

mPAP, mean pulmonary arterial pressure; sPAP, pulmonary arterial systolic pressure; dPAP, pulmonary arterial diastolic pressure; PAPP, pulmonary arterial pulse pressure; PVR, pulmonary vascular resistance; CVP, central venous pressure in the superior vena cava; PAWP, pulmonary artery wedge pressure; CO, cardial output; CI, cardial Index; SV, stroke volume; Cp, pulmonary arterial compliance.

### PAD

**Figure 3:**
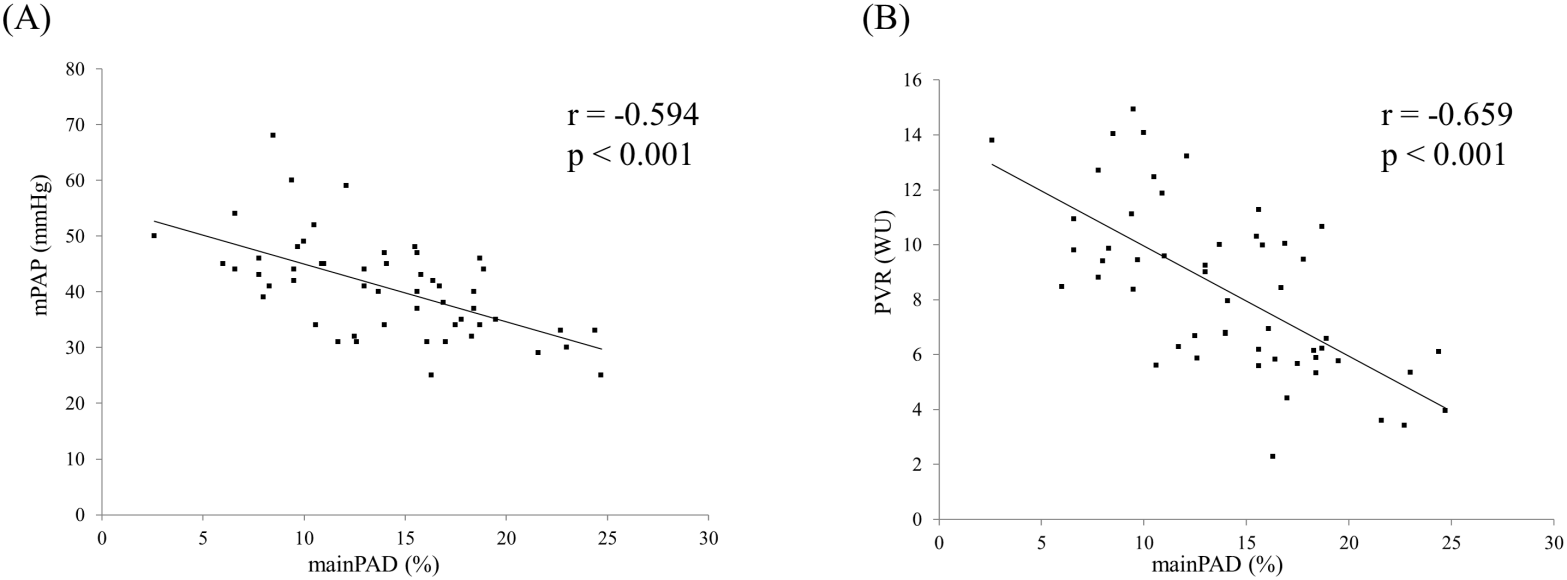
Relation of pulmonary hemodynamics to main pulmonary arterial distensibility (n = 53). mPAP, mean pulmonary arterial pressure; PVR, pulmonary vascular resistance; MainPAD, main pulmonary arterial distensibility.

PAD of the mainPA, rtPA, and ltPA showed a trend toward gradual reduction (Table 3); thus, PAD was largest in the mainPA, followed by the rtPA, and then the ltPA, as follows: mainPA (14.0±5.0%), rtPA (12.8±5.6%), and ltPA (9.7±4.6%). The mainPAD and rtPAD correlated with mPAP, sPAP, dPAP, PAPP, PVR, CO, CI, SV; and Cp. The ltPAD correlated with mPAP, sPAP, dPAP, PVR, SV, and Cp. Of these, good correlations existed between mainPAD and both mPAP (r = −0.594) and PVR (r = −0.659) (P<0.001) (Table 4) (Figure 3).

**Table 3:** CT measurements of patients with CTEPH (n = 53).

CT Parameter	Mean ± SD	Median (min–max)
MainPA-CSA max (cm^2^)	11.9±4.1	10.8 (6.6–27.4)
MainPA-CSA min (cm^2^)	10.3±3.5	9.5 (5.6–22.6)
RtPA-CSA max (cm^2^)	7.8±3.8	7.2 (4.0–31.0)
RtPA-CSA min (cm^2^)	6.9±3.7	6.3 (3.2–29.1)
LtPA-CSA max (cm^2^)	5.8±1.8	5.5 (2.6–13.3)
LtPA-CSA min (cm^2^)	5.2±1.7	5.1 (2.3–12.5)
MainPAD (%)	14.0±5.0	14.0 (2.6–24.6)
RtPAD (%)	12.8±5.6	12.7 (1.2–28.4)
LtPAD (%)	9.7±4.6	9.3 (0.8–22.2)

MainPA, main pulmonary artery; RtPA, right pulmonary artery; LtPA, left pulmonary artery; CSA, cross sectional area; MainPAD, main pulmonary arterial distensibility; RtPAD, right pulmonary arterial distensibility; LtPAD, left pulmonary arterial distensibility.

**Table 4:** Correlation between hemodynamic data and CT measurements (n = 53).

Variables	mPAP	sPAP	dPAP	PAPP	PVR	CO	CI	SV	Cp
MainPAD	**r = −0.594**	**τ = −0.363**	**r = −0.553**	**r = −0.324**	**r = −0.659**	**τ = 0.361**	**τ = 0.202**	**τ = 0.295**	**τ = 0.366**
	**p<0.001**	**p<0.001**	**p<0.001**	**p = 0.018**	**p<0.001**	**p = 0.008**	**p = 0.033**	**p = 0.002**	**p<0.001**
RtPAD	**r = −0.496**	**τ = −0.361**	**r = −0.320**	**r = −0.416**	**r = −0.539**	**τ = 0.396**	**τ = 0.260**	**τ = 0.258**	**τ = 0.338**
	**p<0.001**	**p<0.001**	**p = 0.020**	**p = 0.002**	**p<0.001**	**p = 0.003**	**p = 0.006**	**p = 0.006**	**p<0.001**
LtPAD	**r = −0.369**	**τ = −0.222**	**r = −0.285**	**r = −**0.240	**r = −0.433**	τ = 0.250	τ = 0.144	**τ = 0.179**	**τ = 0.200**
	**p = 0.007**	**p = 0.025**	**p = 0.038**	p = 0.084	**p = 0.001**	p = 0.071	p = 0.129	**p = 0.059**	**p = 0.034**

mPAP, mean pulmonary arterial pressure; sPAP, systolic pulmonary arterial pressure; dPAP, diastolic pulmonary arterial pressure; PAPP, pulmonary arterial pulse pressure; PVR, pulmonary vascular resistance; CO, cardial output; CI, cardial index; SV, stroke volume; Cp, pulmonary arterial compliance; MainPA, main pulmonary artery; RtPA, right pulmonary artery; LtPA, left pulmonary artery; MainPAD, main pulmonary arterial distensibility; RtPAD, right pulmonary arterial distensibility; LtPAD, left pulmonary arterial distensibility.

Significant correlations shown in bold font.

### Clinical worsening analysis after sildenafil

**Figure 4:**
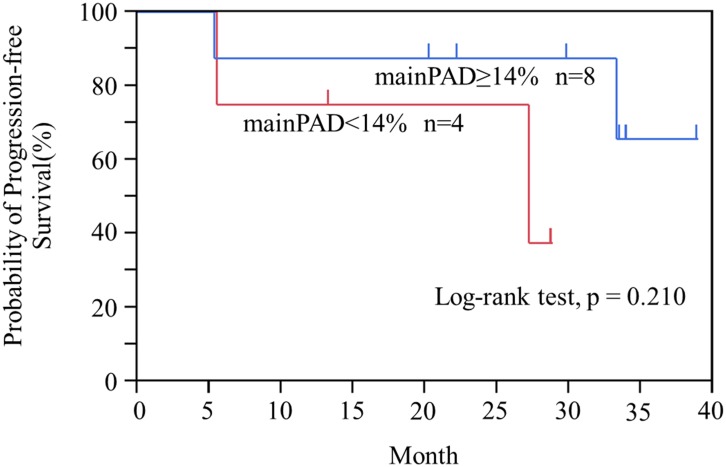
Probability of progression-free survival with mainPAD ≥14% vs mainPAD <14%. MainPAD, main pulmonary arterial distensibility.

Time to clinical worsening was slightly longer in the higher main PAD group (≥14%) than in lower mainPAD group (<14%) (log-rank, p = 0.210) (Figure 4). When the rtPAD and ltPAD were used, similar results were observed.

### Reproducibility

Absolute intra-observer agreement for mainPAD, rtPAD, and ltPAD was a mean difference of −0.25±0.30%, −0.06±0.20%, and −0.45±0.25%, respectively; the agreement range was 0.36 to −0.84%, 0.34 to −0.46%, and 0.05 to −0.95%, respectively. Absolute inter-observer agreement for mainPAD, rtPAD, and ltPAD was a mean difference of −0.09±0.47%, −0.37±0.48%, and 0.48±0.51%, respectively; the agreement range was 0.86 to −1.05%, 0.60 to −1.32%, and 1.52 to −0.55%, respectively.

## Discussion

To the best of our knowledge, this is the first study to assess the ability of 320-slice MDCT to evaluate PAD of each pulmonary artery in subjects with CTEPH. There are two main findings. First, PAD assessed via ECG-gated 320-slice MDCT correlated significantly with mPAP, sPAP, dPAP, PAPP, PVR, CO, CI, SV, and Cp in subjects with CTEPH. Second, the mainPA is suitable for PAD measurements.

### Distensibility

The pathophysiological process of CTEPH includes both pulmonary arterial obstruction and the incomplete resolution and organization of pulmonary thromboemboli. However, it also includes vessel remodeling, the release of vasoconstrictive substances, and reflex vasoconstriction over time. This process results in vasculopathy of precapillary vessels similar to that seen in patients with idiopathic pulmonary arterial hypertension [15], [16]. The distensibility of the major arteries is determined by the elastic properties of elastic and collagen fibers, which are the major constituents of the arterial wall [5], [7], [17]–[19]. When the vessel wall is stretched by higher pressure, the less distensible collagen fibers cause increased stiffness [9]. PAD was primarily measured by CT or MRI, and reflected the degree of blood vessel remodeling. In the normally compliant vessel, the range is between 20% and 25% of the minimal diameter, without significant age variation [20]. Previous reports showed that PAD was reduced in subjects with PH compared to normal cases and correlates with prognosis, exercise tolerance, and pulmonary hemodynamic data in subjects with PH [8], [20]. According to a report by Patz et al., normal MRI-based PAD values in 9 healthy individuals were as follows: mainPAD 25.6±10.7%, rtPAD 21.4±10.7%, and ltPAD 24.5±7.8% [21]. The PAD of each pulmonary artery in this study was lower than found in previous reports. Further, PAD decreased in the following order: mainPA, rtPA, and ltPA; the correlation with mPAP and PVR deteriorated in the same order. We also evaluated the correlation of PAD with pulmonary hemodynamics in 42 cases after excluding 11 cases in which the thrombus was deposited at the measurement site (both sides, 3 cases, right side, 8 cases). Some of the correlation coefficients improved for both rtPAD and ltPAD with pulmonary hemodynamics. However, the correlation between the mainPAD and pulmonary hemodynamics remained superior. Thrombi are less likely to adhere to the mainPA; therefore, the mainPA can evaluate the true PAD. Further, the laterality of blood flow and remodeling of blood vessels together with the difference in thrombotic obstruction between rtPA and ltPA could exist in CTEPH. Therefore, the mainPA is suitable for PAD mesurements.

Toshner et al. reported that a relative area change (RAC) in the rtPA which is calculated using the following equation, and expressed as percentage variation: RAC = [(CSAmaximum−CSAminimum)/CSAminimum]×100, correlates with the functional response to sildenafil, the 6-minites walk test (6MWT), N-terminal pro-brain natriuretic peptide (NT pro-BNP), and WHO functional class [22]. In this study, 13 patients were administered sildenafil after measuring the PAD; the PAD was not associated with improvement in either the 6MWT or brain natriuretic peptide (BNP) at a mean of 4.6±1.9 months (range, 2–9 months) after the induction of sildenafil. However, time to clinical worsening, indicated by death or hospitalization due to right heart failure, was slightly longer in the higher mainPAD group than in lower mainPAD group. Further studies are needed to clarify the association of distensibility with response to sildenafil or other drugs. In addition, Mahapatra et al. reported that Cp is a strong predictor of mortality in PAH [23], and Lankhaar et al. reported that Cp strongly contributes to right ventricular afterload [24]. PAD was significantly correlated with Cp in this study. Thus, PAD may be a prognostic indicator.

MDCT has advantages over MRI for PAD evaluation. Using ECG-gated 320-slice MDCT, all cross-sections in the pulmonary artery cine images can be observed, even after imaging. Using MRI, it is necessary to select a cross-section to view before imaging; this requirement presents a limitation, as the pulmonary artery deforms easily and moves during the cardiac cycle. In addition, the breath-hold time was no more than 10 s with MDCT, which would be beneficial in patients with CTEPH who may be unable to perform the extended breath hold required for MRI [13]. Thus, CT could be superior to MRI for pulmonary artery evaluation in patients with CTEPH. However, caution must be exercised in choosing CT due to radiation exposure, even though the radiation burden with 320-slice volume CT is lower than that with helical CT, as it avoids overlapping rotations [13]. In the present study, the total radiation dose was approximately 10−20 millisieverts. In addition, CT also allowed us to evaluate coronary artery disease and thus assess the necessity of simultaneous coronary artery graft bypass surgery during pulmonary endarterectomy as a substitute for conventional invasive coronary angiography [12], [25]. Such benefits must be weighed against the potential risk from radiation exposure.

### Study limitations

This study had some limitations. First, this single-center retrospective study included a small number of subjects. However, because the disease is rare, the present report could be noteworthy within this field. Second, the correlation between enhanced ECG-gated 320-slice MDCT parameters and RHC pulmonary hemodynamic data should ideally be studied by performing the two examinations on the same day; in our study, most patients underwent the two examinations within 16 days of each other.

### Conclusions

PAD measured by enhanced ECG-gated 320-slice MDCT correlated with mPAP, sPAP, dPAP, PVR, CO, CI, SV and Cp in subjects with CTEPH. The mainPA is suitable for measuring of PAD.
